# High SEMA4C expression promotes the epithelial-mesenchymal transition and predicts poor prognosis in colorectal carcinoma

**DOI:** 10.18632/aging.104038

**Published:** 2020-11-07

**Authors:** Yufang Hou, Weiqi Wang, Zifan Zeng, Wenqiang Gan, Silin Lv, Tiegang Li, Zheng Yan, Rixin Zhang, Min Yang

**Affiliations:** 1 State Key Laboratory of Bioactive Substances and Function of Natural Medicine, Institute of Materia Medica, Chinese Academy of Medical Sciences and Peking Union Medical College, Beijing 100050, China

**Keywords:** colorectal cancer, prognosis, prediction, consensus molecular subtype, epithelial-mesenchymal transition

## Abstract

Semaphorin 4C (SEMA4C), is an important regulator of axonal guidance and aggravates tumor development. However, the roles and prognostic value of SEMA4C in colorectal cancer (CRC) remain unclear. Here, bioinformatics analyses of transcriptome data from multiple CRC patient datasets and immunohistochemical staining of a CRC tissue microarray (TMA) (n=83) showed that SEMA4C mRNA and protein expression were higher in CRC tissues than normal colorectal tissues. SEMA4C mRNA and protein expression correlated with pathologic stage and metastasis in CRC patients. Higher SEMA4C expression was associated with shorter overall survival, consensus molecular subtype 4 (CMS4), and DNA hypomethylation of SEMA4C in CRC patients. Multivariate Cox regression analyses revealed that SEMA4C expression was an independent prognostic predictor in CRC patients. Gene set expression analysis (GSEA) illustrated that SEMA4C expression had remarkable correlations with epithelial-mesenchymal transition (EMT) as well as hedgehog, Wnt/β-catenin, TGF-β, and Notch signaling pathways. Receiver operating characteristic (ROC) curve analysis demonstrated that SEMA4C expression accurately distinguished between the CMS4 and CMS1-3 subtypes of CRC patients. By inhibiting EMT, SEMA4C silencing reduced *in vitro* proliferation, migration, and invasion by CRC cells. These findings suggest that SEMA4C is a CMS4-associated gene that enhances CRC progression by inducing EMT.

## INTRODUCTION

Colorectal cancer (CRC) is the third leading cause of cancer-related deaths and fourth most commonly diagnosed cancer worldwide [1, 2]. The survival rates of CRC patients have remained low despite advances in surgical techniques and perioperative care, neoadjuvant chemotherapy, radiotherapy and molecular targeted therapy. This is because most patients are diagnosed in advanced stages and show high rates of recurrence [3–5]. There is thus an urgent need for highly sensitive and accurate prognostic biomarkers. Recently, a new classification system was developed for CRC patients based on consensus molecular subtypes (CMS). CRC patients were subdivided into four molecular subtypes based on their distinct biological characteristics. The CMS1 subtype is characterized by microsatellite instability and strong immune activation. The CMS2 subtype is characterized by upregulation of WNT and MYC signaling pathways. The CMS3 subtype is characterized by metabolic dysregulation. And the CMS4 subtype is characterized by a mesenchymal phenotype, transforming growth factor-β (TGF-β) activation, stromal invasion, and neoangiogenesis [6, 7]. These CMS subtypes can potentially be used for risk stratification, targeted therapeutic strategy, and prognostic prediction of survival outcomes [8, 9]. However, clinical implementation of CMS subtypes has been technically challenging as yet.

The semaphorins are a large family of secreted and membrane-associated proteins that are involved in axon guidance, cellular differentiation, cytoskeletal rearrangement, and cell motility [10]. Semaphorin family members also regulate proliferation, angiogenesis, and immune responses of tumor cells [11–13]. Semaphorin 4C (SEMA4C) is highly expressed in breast [14], lung [15], cervical [16] and liver [17] cancers. SEMA4C is involved in oncogenic signaling and is a potential therapeutic target for invasive breast cancer [14, 18], hepatocellular carcinoma [17], glioma [19] and osteosarcoma [20]. Moreover, SEMA4C overexpression promotes cellular proliferation, migration and epithelial-mesenchymal transition (EMT) in breast cancer and non-small cell lung cancer [15, 21–23]. However, the prognostic significance of SEMA4C has not been established in patients with CRC. Therefore, in the present study, we analyzed the expression of SEMA4C in CRC tissues from multiple public databases and investigated its prognostic significance as well as the molecular mechanisms.

## RESULTS

### SEMA4C expression is altered in different cancer types including CRC

**Figure 1:**
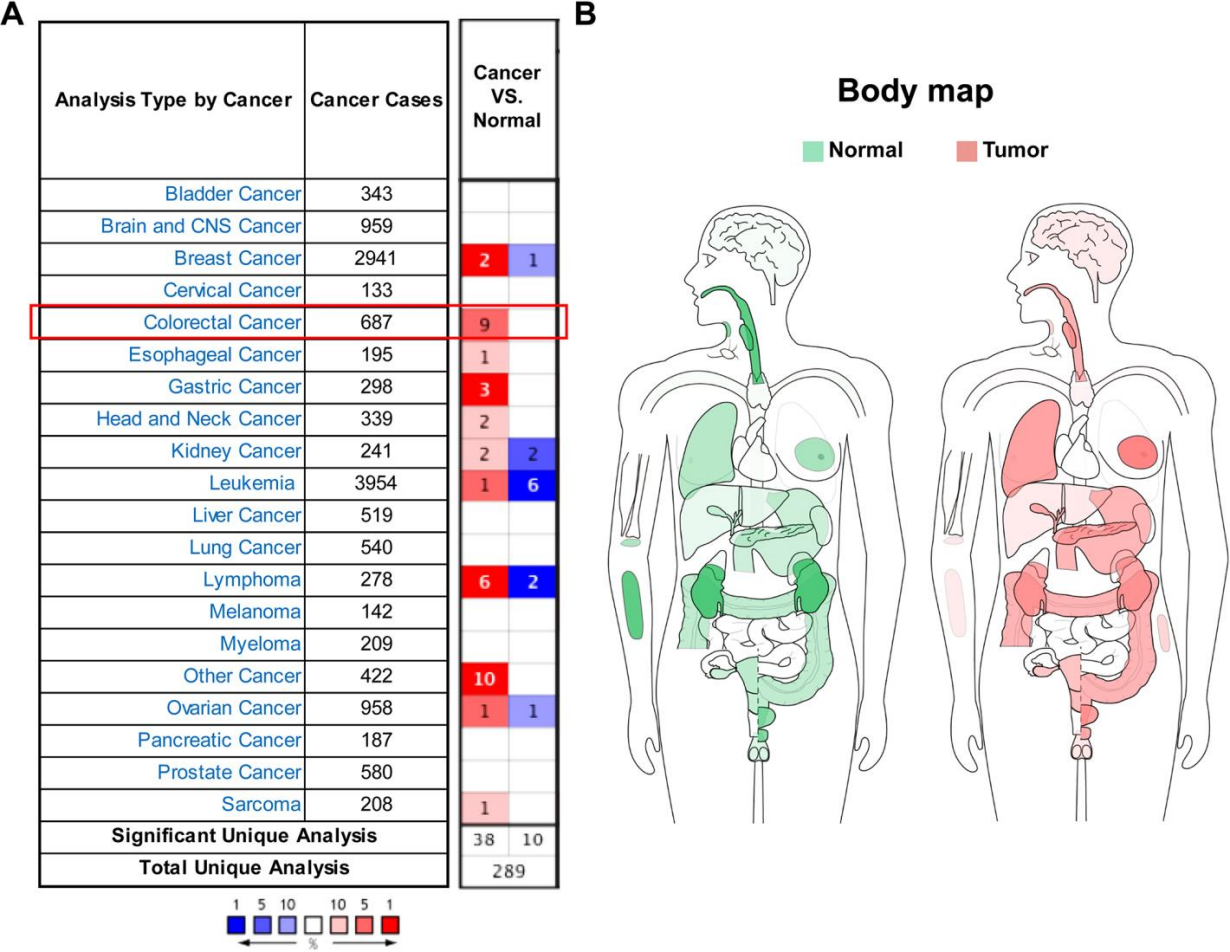
**SEMA4C mRNA expression is altered in different human cancers.** (**A**) Oncomine database analysis results of SEMA4C mRNA levels in the tumor and normal tissues in human cancers. Note: Red and blue denote upregulation and downregulation of SEMA4C in the tumor tissues, respectively. (**B**) Interactive human body map constructed using the GEPIA webserver shows the status of SEMA4C mRNA expression in the tumor (red) and normal (green) tissue samples from different cancer types based on the TCGA data.

Oncomine database analysis showed that SEMA4C mRNA levels were significantly upregulated in colorectal, gastric, head and neck, esophageal cancers, and sarcoma compared to the corresponding normal tissues. While conflicting evidences of dysregulation were found in some other cancers like breast cancer and kidney cancer (Figure 1A). SEMA4C mRNA expression profile in different human tissues is shown in the interactive human body map constructed using the Gene Expression Profiling Interactive Analysis (GEPIA) database (Figure 1B). Tumor Immune Estimation Resource (TIMER) database analysis of SEMA4C mRNA expression in various cancer types is shown in Supplementary Figure 1. Overall, these results demonstrate that SEMA4C mRNA levels are significantly altered in the tumor tissues compared to the corresponding normal tissues (Figure 1 and Supplementary Figure 1).

**Figure 2:**
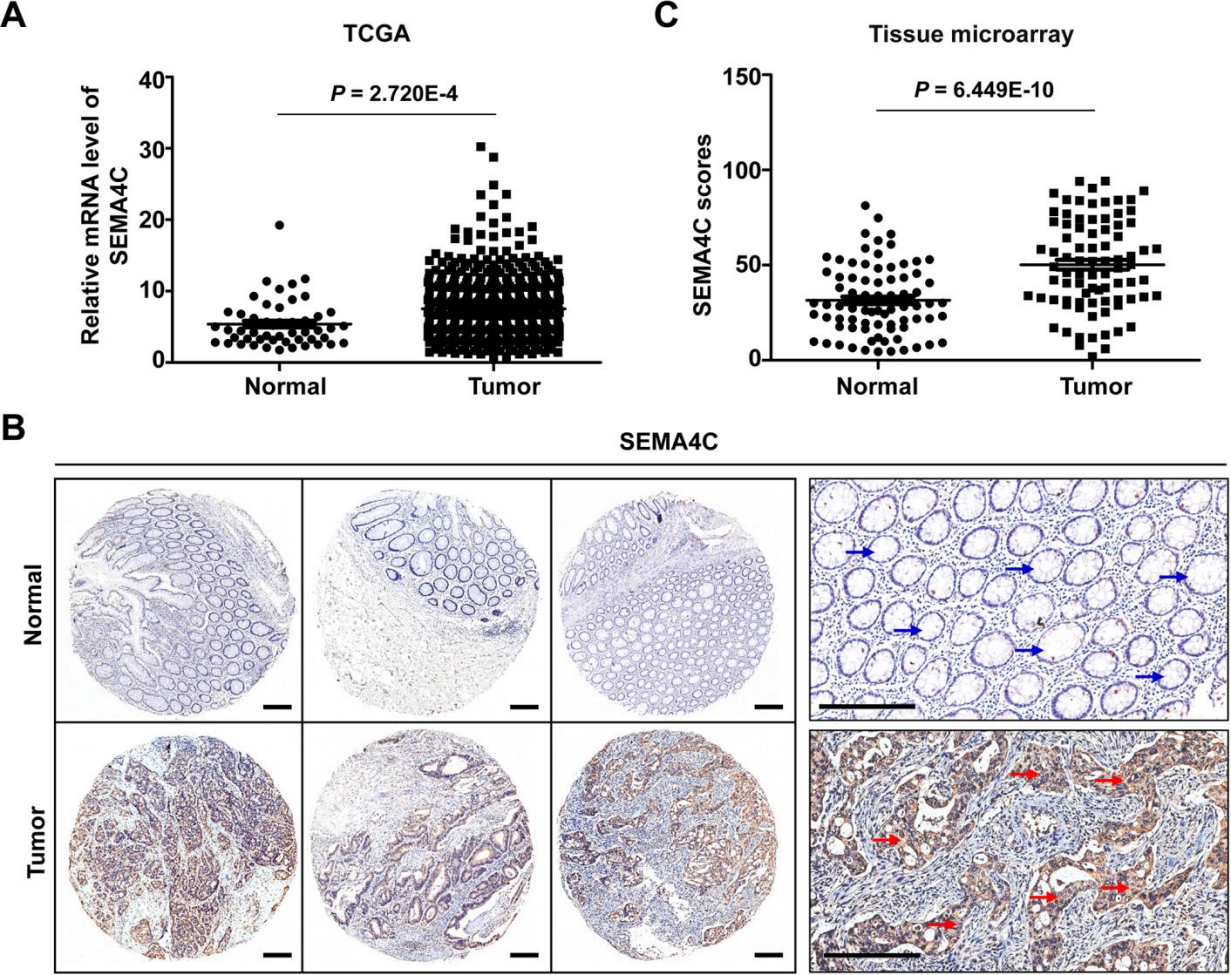
**SEMA4C mRNA and protein levels are upregulated in the CRC tissues.** (**A**) The relative SEMA4C mRNA expression level in normal colorectal and CRC tissues from the TCGA-COAD and TCGA-READ datasets. (**B**) Representative micrographs show SEMA4C immunohistochemical staining of the 83 pairs of colon cancer and adjacent normal colon tissue samples in the tissue microarray (TMA). Note: Scale bars=200 μm; red arrow indicates strong positive SEMA4C protein expression in the colon cancer tissue; blue arrow indicates negative SEMA4C protein expression in the adjacent normal tissue. (**C**) Quantitative analysis of SEMA4C protein expression scores based on the immunohistochemical staining of the TMA with 83 pairs of colon carcinoma and adjacent normal colon tissue samples.

RNA-sequencing data analysis of the The Cancer Genome Atlas (TCGA)-CRC dataset (597 CRC and 51 normal colorectal samples) showed that the SEMA4C mRNA levels were significantly upregulated in the CRC tissues (*P* = 2.720E-4; Figure 2A). Furthermore, immunohistochemical analysis of a tissue microarray (TMA) with 83 matched colon cancer and adjacent normal colon tissues showed that SEMA4C protein levels were significantly upregulated in the CRC tissues (*P* = 6.449E-10); SEMA4C protein was mainly localized to the cell membrane and cytoplasm in the CRC cells (Figure 2B, 2C).

### SEMA4C expression correlates with pathologic stage and metastasis in CRC patients

We then analyzed the association between SEMA4C expression and the clinicopathological characteristics of CRC patients using the TCGA-CRC dataset (597 CRC and 51 normal colorectal samples). As shown in Table 1, SEMA4C mRNA expression was significantly associated with the histological tumor type (*P* = 0.019), pathologic stage (*P* = 0.008), lymph node metastasis (*P* = 0.011), distant metastasis (*P* = 0.013), microsatellite instability (MSI) status (*P* = 0.038) and CMS subtypes (*P* = 2.091E-9). However, SEMA4C mRNA levels were not associated with clinicopathological characteristics such as age, gender, race, T stage, venous invasion, and pre-treatment serum carcinoembryonic antigen (CEA) levels. We also analyzed the relationship between SEMA4C protein expression levels in CRC specimens based on the immunohistochemical staining data from the TMA and the clinicopathological parameters of RC patients. As shown in Table 2, SEMA4C protein expression positively correlated with pathologic stage (*P* = 0.047), T stage (*P* = 0.010) and distant metastasis (*P* = 0.033). These results suggest that SEMA4C mRNA and protein expression correlates with pathologic stage and metastasis in CRC patients.

**Table 1:** The association between SEMA4C mRNA levels and the clinicopathological variables in the TCGA-CRC dataset.

**Variables**	**N**	**Percent**	**SEMA4C Mean ± SD**	**ANOVA *P* value**
Age				0.236
≤ 69	339	56.784	7.688 ± 4.210	
> 69	258	43.216	7.292 ± 3.805	
Gender				0.124
Male	323	54.104	7.282 ± 3.789	
Female	274	45.896	7.793 ± 4.311	
Race				0.533
White	290	48.576	7.592 ± 3.656	
Black or African American	64	10.720	8.161 ± 4.539	
Asian	12	2.010	8.095 ± 4.811	
Location				0.457
Colon	438	73.367	7.443 ± 4.033	
Rectum	159	26.633	7.721 ± 4.071	
Histology type				0.019
I (adenocarcinoma)	513	85.930	7.668 ± 4.017	
II (mucinous adenocarcinoma)	73	12.228	6.481 ± 4.097	
Pathologic stage				0.008
1	103	17.253	7.127 ± 3.951	
2	218	36.516	7.030 ± 3.849	
3	175	29.313	7.703 ± 4.172	
4	86	14.405	8.686 ± 4.113	
Pathologic T stage				0.157
T1	20	3.350	6.671 ± 5.648	
T2	103	17.253	7.107 ± 3.879	
T3	408	68.342	7.503 ± 3.960	
T4	64	10.720	8.428 ± 4.098	
Pathologic N stage				0.011
N0	338	56.616	7.081 ± 3.949	
N1	146	24.456	7.914 ± 4.272	
N2	110	18.425	8.243 ± 3.855	
Pathologic M stage				0.013
M0	443	74.204	7.368 ± 4.047	
M1	84	14.070	8.581 ± 4.179	
Venous invasion				0.196
No	394	65.997	7.327 ± 3.890	
Yes	125	20.938	7.828 ± 3.345	
MSI				0.038
MSS	413	69.179	7.769 ± 4.002	
MSI	181	30.318	7.024 ± 4.070	
CMS				2.091E-9
1	73	12.228	6.189 ± 3.854	
2	209	35.008	7.798 ± 4.336	
3	72	12.060	5.751 ± 2.821	
4	137	22.948	9.077 ± 3.804	
Pretreatment CEA level				0.941
≤ 10	296	49.581	7.671 ± 4.210	
> 10	89	14.908	7.709 ± 4.241	

**Table 2:** The association between SEMA4C protein levels and the clinicopathological variables in the TMA cohort of colon cancer patients.

**Variables**	**N**	**Percent**	**SEMA4C Mean ± SD**	**ANOVA *P* value**
Age				0.963
≤ 59	43	51.807	50.356 ± 24.529	
> 59	40	48.193	50.113 ± 23.313	
Gender				0.664
Male	49	59.036	51.191 ± 21.799	
Female	34	40.964	48.867 ± 26.714	
T				0.010
T1+2	17	20.482	37.130 ± 21.067	
T3+4	66	79.518	53.616 ± 23.433	
Lymphatic metastasis				0.146
No	51	61.446	47.220 ± 23.150	
Yes	32	38.554	55.050 ± 24.406	
Distant metastasis				0.033
No	45	54.217	45.137 ± 22.922	
Yes	38	45.783	56.281 ± 23.705	
Pathologic stage				0.047
1	12	14.458	34.517 ± 20.757	
2	18	21.687	49.854 ± 22.545	
3	15	18.072	47.972 ± 23.700	
4	38	45.783	56.281 ± 23.705	
Pretreatment CEA				0.167
Low	55	66.265	47.651 ± 23.884	
High	28	33.735	55.322 ± 23.231	
Pretreatment CA199				0.354
Low	60	72.289	48.729 ± 22.920	
High	23	27.711	54.177 ± 26.094	

### High SEMA4C expression predicts poor prognosis of CRC patients

**Figure 3:**
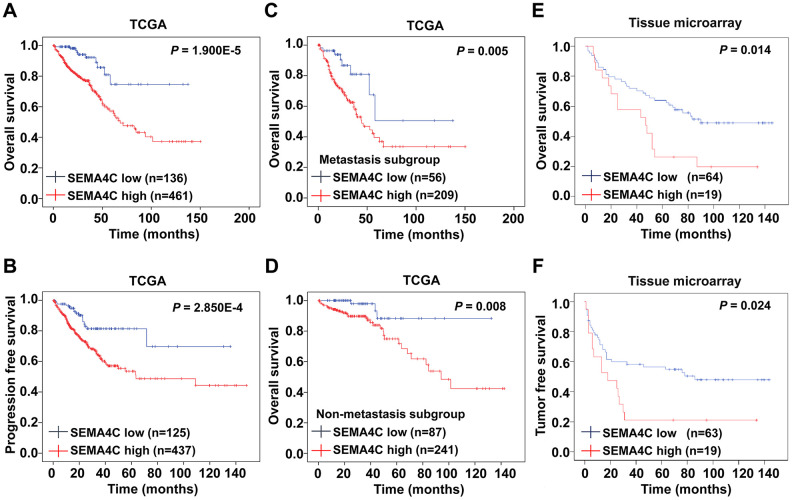
**High SEMA4C expression correlates with poor survival outcomes in CRC patients.** (**A**, **B**) Kaplan-Meier survival curves show OS and PFS of high- and low-SEMA4C expressing CRC patients from the TCGA database. (**C**, **D**) Kaplan-Meier survival curves show the OS of high- and low-SEMA4C expressing CRC patients belonging to the metastatic and non-metastatic subgroups. (**E**, **F**) Kaplan-Meier survival curves show OS and TFS of high- and low-SEMA4C expressing CRC patients from the TMA-colon cancer cohort.

Next, we analyzed the prognostic value of SEMA4C in CRC patients. We divided the TCGA-CRC patients into high (n=461) and low SEMA4C expression groups (n=136) and evaluated their survival outcomes. Kaplan-Meier survival curve analysis showed that overall survival (OS) and progression-free survival (PFS) of the SEMA4C high expression group was significantly shorter than the SEMA4C low expression group (OS: *P* = 1.900E-5; PFS: *P* = 2.850E-4; Figure 3A, 3B). Besides, high SEMA4C expression correlated with significantly shorter OS in CRC patients with lymph node and distant metastasis (*P* = 0.005; Figure 3C) and CRC patients without lymph node and distant metastasis (*P* = 0.008; Figure 3D). Furthermore, CRC patients with high SEMA4C protein expression in the TMA-CRC cohort also showed poorer OS (*P* = 0.014) and tumor-free survival (TFS; *P* = 0.024) compared to those with low SEMA4C expression (Figure 3E, 3F).

We then performed univariate and multivariate Cox regression analysis to identify risk factors that predict prognosis of CRC patients. Univariate Cox regression analysis showed that age, pathologic T stage, pathologic N stage, pathologic M stage, pretreatment CEA levels, venous invasion and SEMA4C expression correlated with the OS of CRC patients from the TCGA dataset (*P* < 0.001; Table 3). Furthermore, multivariate Cox regression analysis showed that SEMA4C expression was an independent risk factor for OS in the CRC patients (hazard ratio [HR] = 3.496, 95% confidence interval [CI]: 1.239-9.860, *P* = 0.018; Table 3). In the TMA-CRC cohort, univariate Cox regression model showed that T stage, lymphatic metastasis, distant metastasis, pretreatment CEA levels, pretreatment CA199 levels, and SEMA4C levels were associated with the OS of CRC patients (*P* < 0.05; Table 4). Multivariate analysis of the TMA-CRC cohort showed that T stage, distant metastasis and pretreatment CA199 levels were the independent prognostic factors for OS in colon cancer patients (*P* < 0.05; Table 4). However, SEMA4C expression did not show statistical significance in the multivariate analysis (*P* > 0.05; Table 4). Overall, our findings suggest that SEMA4C expression is an independent prognostic indicator for patients with CRC.

**Table 3:** Univariate and multivariate Cox regression analysis of the prognostic significance of SEMA4C mRNA expression and other clinicopathogical parameters in TCGA-CRC patients.

**Variables**	**Univariate analysis**		**Multivariate analysis**	
	**HR (95% CI)**	***P* value**	**HR (95% CI)**	***P* value**
Age				
> 69 VS. ≤ 69	2.107 (1.468, 3.025)	< 0.001	3.104 (1.735, 5.551)	< 0.001
Gender				
Female VS. Male	0.931 (0.653, 1.327)	0.691		
Race				
Black VS. White	1.046 (0.588, 1.858)	0.879		
Type				
Rectum VS. Colon	0.807 (0.523, 1.245)	0.332		
Histology type				
II VS. I	1.411 (0.855, 2.330)	0.178		
Pathologic T		< 0.001		0.168
T2 VS. T1	0.954 (0.202, 4.500)	0.952	0.624 (0.064, 6.040)	0.684
T3 VS. T1	2.039 (0.502, 8.290)	0.319	0.812 (0.106, 6.197)	0.841
T4 VS. T1	6.752 (1.600, 28.491)	0.009	1.815 (0.217, 15.168)	0.582
Pathologic N		< 0.001		0.555
N1 VS. N0	1.920 (1.222, 3.017)	0.005	1.502 (0.604, 3.734)	0.381
N2 VS. N0	4.028 (2.661, 6.098)	< 0.001	1.730 (0.633, 4.728)	0.285
Pathologic M				
M1 VS. M0	4.491 (3.013, 6.695)	< 0.001	3.069 (1.482, 6.358)	0.003
SEMA4C				
High VS. low	3.726 (1.952, 7.112)	< 0.001	3.496 (1.239, 9.860)	0.018
Venous invasion				
Yes VS. No	2.368 (1.603, 3.499)	< 0.001	2.517 (1.376, 4.604)	0.003
MSI				
MSI VS. MSS	1.156 (0.798, 1.675)	0.443		
CMS		0.278		
CMS2 VS. CMS1	0.847 (0.473, 1.517)	0.577		
CMS3 VS. CMS1	0.700 (0.336, 1.456)	0.340		
CMS4 VS. CMS1	1.221 (0.672, 2.218)	0.512		
Pretreatment CEA				
> 10 VS. ≤ 10	2.941 (1.742, 4.964)	< 0.001	2.270 (1.124, 4.585)	0.022

**Table 4:** Univariate and multivariate Cox regression analysis of the prognostic significance of SEMA4C protein expression and other clinicopathological parameters in the TMA cohort of colon cancer patients.

**Variables**	**Univariate analysis**		**Multivariate analysis**	
	**HR (95% CI)**	***P* value**	**HR (95% CI)**	***P* value**
Age				
> 59 VS. ≤ 59	1.424 (0.797, 2.546)	0.233		
Gender				
Male VS. Female	1.037 (0.576, 1.868)	0.903		
T				
T3+4 VS. T1+2	4.277 (1.526, 11.985)	0.006	4.061 (1.339, 12.320)	0.013
Lymphatic metastasis				
Yes VS. No	1.987 (1.112, 3.551)	0.020	1.245 (0.687, 2.256)	0.470
Distant metastasis				
Yes VS. No	16.247 (7.255, 36.385)	<0.001	16.418 (7.010, 38.451)	<0.001
CEA				
High VS. Low	2.806 (1.551, 5.076)	0.001	1.879 (0.968, 3.648)	0.062
CA199				
High VS. Low	2.950 (1.621, 5.369)	<0.001	2.047 (1.065, 3.936)	0.032
SEMA4C				
High VS. Low	2.136 (1.147, 3.977)	0.017	0.814 (0.414, 1.599)	0.550

### Functional enrichment analyses show that SEMA4C promotes EMT in CRC tissues

**Figure 4:**
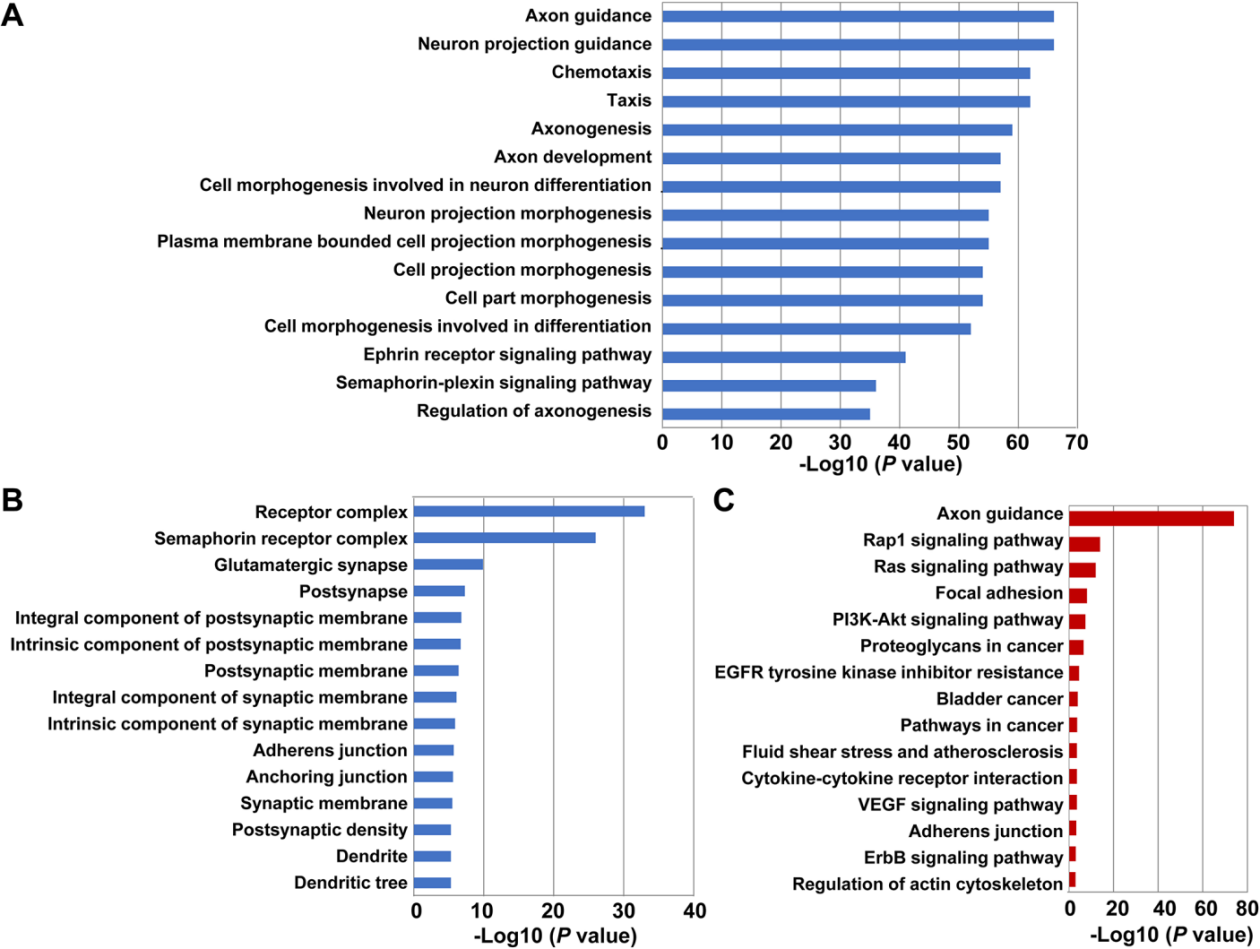
**Functional enrichment analysis of GO terms and KEGG pathways related to SEMA4C and 50 SEMA4C-associated genes in CRC tissues.** (**A**, **B**) The GO terms for the enriched (**A**) biological functions, and (**B**) cellular components related to SEMA4C and 50 SEMA4C-associated genes in the CRC tissues are shown. (**C**) The enriched KEGG pathways related to SEMA4C and 50 SEMA4C-associated genes in the CRC tissues are shown.

We then investigated the potential mechanisms associated with SEMA4C expression. We identified SEMA4C-related genes using the STRING online database and generated a protein-protein interaction (PPI) network of SEMA4C and 50 SEMA4C-associated genes (nodes) including 810 inter-genic interactions (edges) using the Cytoscape software (Supplementary Figure 2). The genes with the highest degree of association with SEMA4C included the ras homolog family member A (RHOA), ephrin A2 (EFNA2), ephrin A3 (EFNA3), ephrin A4 (EFNA4), ephrin B1 (EFNB1) and ephrin B3 (EFNB3), all of which are related to cell migration and cell adhesion (Supplementary Figure 2). We then performed Gene Ontology (GO) and Kyoto Encyclopedia of Genes and Genomes (KEGG) pathway analyses of SEMA4C and the 50 SEMA4C-associated genes to determine the biological processes and pathways activated by SEMA4C. The top 15 significantly enriched GO terms related to biological processes and cellular components, and KEGG pathways are shown in Figure 4. We found that the significantly enriched GO terms related to biological processes included axon guidance, chemotaxis, taxis and cell morphogenesis involved in differentiation, and ephrin receptor signaling (Figure 4A). Meanwhile, the significantly enriched GO terms related to cellular components involved receptor complex, postsynapse, postsynaptic membrane, adherens junction, anchoring junction, and dendrite (Figure 4B). Moreover, the significantly enriched KEGG pathways contained Ras signaling pathway, focal adhesion, phosphatidylinositol 3-kinase (PI3K)-Akt signaling pathway, proteoglycans in cancer, bladder cancer, pathways in cancer, vascular endothelial growth factor (VEGF) signaling pathway, adherens junctions, and erb-b2 receptor tyrosine kinase (ErbB) signaling pathway (Figure 4C). Overall, GO and KEGG pathway analyses demonstrate that SEMA4C promotes CRC progression.

**Figure 5:**
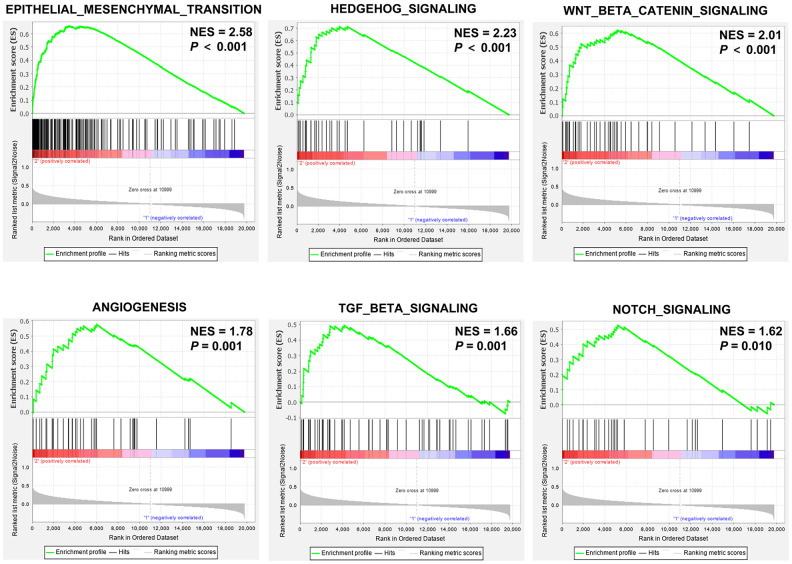
**GSEA enrichment plots of the TCGA-CRC dataset.** GSEA results show the enrichment of gene sets related to epithelial mesenchymal transition (EMT), hedgehog signaling, Wnt/β-catenin signaling, angiogenesis, TGF-β signaling, and notch signaling in CRC patients with high SEMA4C expression. Note: NES, normalized enrichment score.

To gain further insights into the role of SEMA4C in CRC pathogenesis, we performed gene set enrichment analysis (GSEA) of the RNA-sequencing data from the TCGA-CRC dataset. The results showed that gene sets related to EMT (normalized enrichment score [NES] = 2.58), hedgehog signaling (NES = 2.23), Wnt/β-catenin signaling (NES = 2.01), angiogenesis (NES = 1.78), TGF-β signaling (NES = 1.66), and Notch signaling (NES = 1.62) were significantly enriched in CRC patients with high SEMA4C expression (*P* < 0.05; Figure 5).

**Figure 6:**
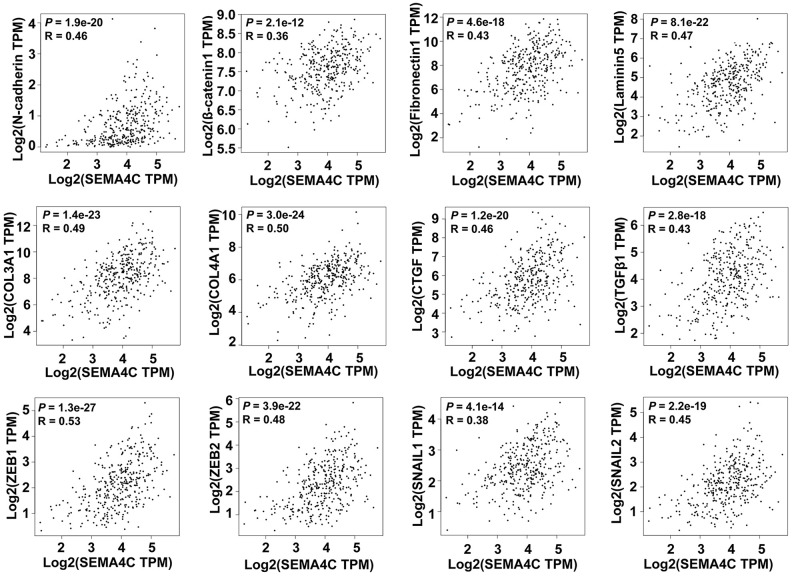
**SEMA4C expression correlates with the expression of EMT-related genes in CRC patients.** Spearman’s correlation analysis results show the association between SEMA4C levels and the expression of EMT-related genes, including N-cadherin, β-catenin, Fibronectin1, Laminin5, COL3A1, COL4A1, CTGF, TGFβ1, ZEB1, ZEB2, SNAIL1, and SNAIL2 in the CRC tissues using the GEPIA database. Note: TPM, transcripts per million; R denotes the Spearman correlation coefficient.

Next, we used the GEPIA database to analyze the correlation between SEMA4C and the EMT-related genes with the TCGA-CRC data. The results showed a strong correlation between SEMA4C and EMT-associated genes, including mesenchymal markers (N-cadherin and β-catenin), extracellular matrix components (fibronectin1, laminin5, collagen type III alpha 1 [COL3A1], and collagen type IV alpha 1 [COL4A1]), pro-fibrogenic factors (connective tissue growth factor [CTGF], TGF-β1), specific transcription factors (zinc finger E-box binding homeobox 1 and 2 [ZEB1 and ZEB2]), snail family transcriptional repressors 1 and 2 [SNAIL1 and SNAIL2]) in the TCGA-CRC cohort (R > 0.3; Figure 6). These findings suggest that SEMA4C may serve as an indicator of EMT in CRC.

### High SEMA4C expression correlates with the CMS4 subtype in the CRC patients

**Figure 7:**
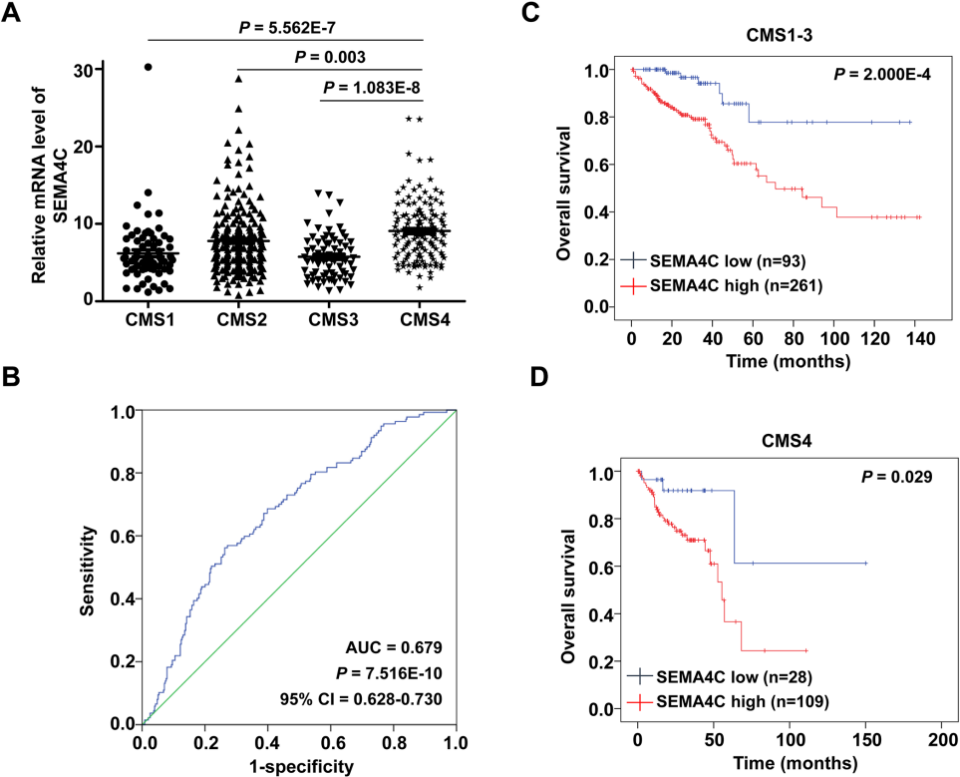
**SEMA4C overexpression correlates with the CMS4 subtype of CRC.** (**A**) Box-scatter plot shows the SEMA4C mRNA level in CMS1, CMS2, CMS3 and CMS4 subtypes of CRC patient tissues in the TCGA dataset. (**B**) ROC curve analysis shows that SEMA4C expression levels accurately discriminate between CMS4 and non-CMS4 molecular subtypes of the TCGA-CRC dataset. Note: AUC: area under receiver operating characteristic, CI: confidence interval. (**C**, **D**) Kaplan-Meier survival curves show the OS in the high- and low- SEMA4C expressing (**C**) CMS1-3 and (**D**) CMS4 subgroups of TCGA-CRC patients. Log-rank test was used to determine the statistical differences between the groups.

Several studies show that among the four CMS subtypes, the CMS4 subtype is related to worse prognosis and upregulation of EMT-associated genes [8, 9]. Thus, we evaluated differences in SEMA4C expression in the TCGA-CRC patients belonging to the four CMS subtypes. The results showed that SEMA4C mRNA expression was significantly higher in the CMS4 subtype CRC patients compared to those belonging to the CMS1-3 subtypes (Figure 7A). Receiver operator characteristic (ROC) curve analysis revealed that the levels of SEMA4C mRNA expression effectively discriminated between CMS4 versus CMS1-3 subtypes in the TCGA-CRC dataset (Figure 7B). Furthermore, Kaplan-Meier survival curve analysis illustrated that patients with higher SEMA4C expression belonging to both CMS1-3 (*P* = 2.000E-4) and CMS4 (*P* = 0.029) subtypes demonstrated shorter OS compared to the corresponding patients with low SEMA4C expression (Figure 7C, 7D).

### SEMA4C expression correlates with the DNA methylation status of SEMA4C gene

**Figure 8:**
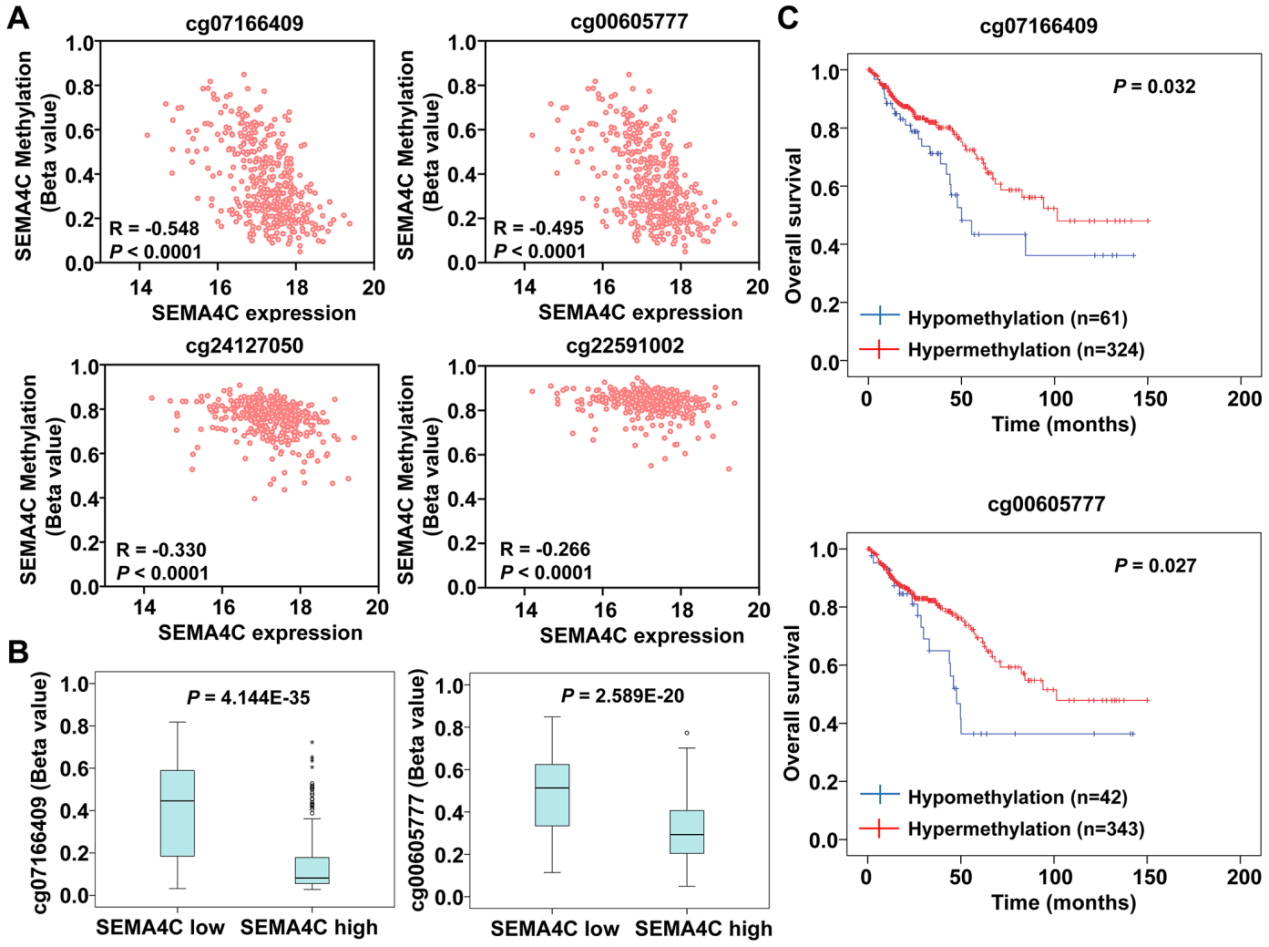
**SEMA4C expression negatively correlates with the DNA methylation status of the SEM4AC gene in the CRC patients.** (**A**) Spearman’s correlation analysis shows the relationship between DNA methylation status of the SEMA4C gene and the expression levels of SEMA4C mRNA in the CRC samples. Note: R denotes the Spearman’s correlation coefficient. (**B**) Boxplots show the methylation levels of the CpG island shore, cg07166409 (left) and cg00605777 (right) in the SEMA4C gene in CRC patients with low- and high-expression of SEMA4C. (**C**) Kaplan-Meier survival curves show the OS of TCGA-CRC patients with hypomethylation (blue line) and hypermethylation (red line) of the SEMA4C gene in the CpG island shore, cg07166409 (above) and cg00605777 (below).

Next, we evaluated the relationship between differential methylation of the SEMA4C gene and SEMA4C mRNA levels in the CRC tissues using the MEXPRESS tool. The methylation status of different regions of the SEMA4C gene in the TCGA-colon adenocarcinoma (TCGA-COAD) and TCGA-rectal adenocarcinoma (TCGA-READ) are shown in Supplementary Figure 3 and 4, respectively. Spearman correlation analysis showed that SEMA4C mRNA expression negatively correlated with the methylation status of the CpG islands, cg24127050 (R = -0.330) and cg22591002 (R =-0.266), in the SEMA4C gene promoter region (Figure 8A). Moreover, SEMA4C mRNA levels negatively correlated with the methylation levels of CpG island shore, cg07166409 (R = -0.548) and cg00605777 (R = -0.495), both of which are located in the first exon of the SEMA4C gene (Figure 8A). Furthermore, the methylation levels of CpG island shore, cg07166409 and cg00605777 in CRC with high SEMA4C expression were significantly lower than those of CRC with low SEMA4C expression (Figure 8B). The overall survival was significantly shorter in CRC patients with hypomethylated CpG island shore, cg07166409 (*P* = 0.032) and cg00605777 (*P* = 0.027) in the SEMA4C gene (Figure 8C).

DNA methylation status involves co-ordinated action of several methyl transferases such as DNA methyltransferase 1 (DNMT1) and DNMT3, and DNA demethylating enzymes such as the ten-eleven translocation (TET) enzymes [24]. Therefore, we investigated the expression of DNA methylating and demethylating enzymes in the CRC tissues. The mRNA expression of DNMT1 and DNMT3B was significantly lower in the CMS4 patients compared to the CMS1-3 patients, whereas, TET1 levels were significantly upregulated in the CMS4 patients (Supplementary Figure 5). Moreover, we did not observe any higher copy number alterations of the SEMA4C gene in the CRC samples (Supplementary Figure 6A). The frequency of SEMA4C mutations was also low and insignificant in the CRC patients (Supplementary Figure 6B). These findings suggest that hypomethylation of the SEMA4C gene upregulates SEMA4C mRNA expression in the CRC patients.

### SEMA4C knockdown inhibits proliferation, migration, and invasion of CRC cells

**Figure 9:**
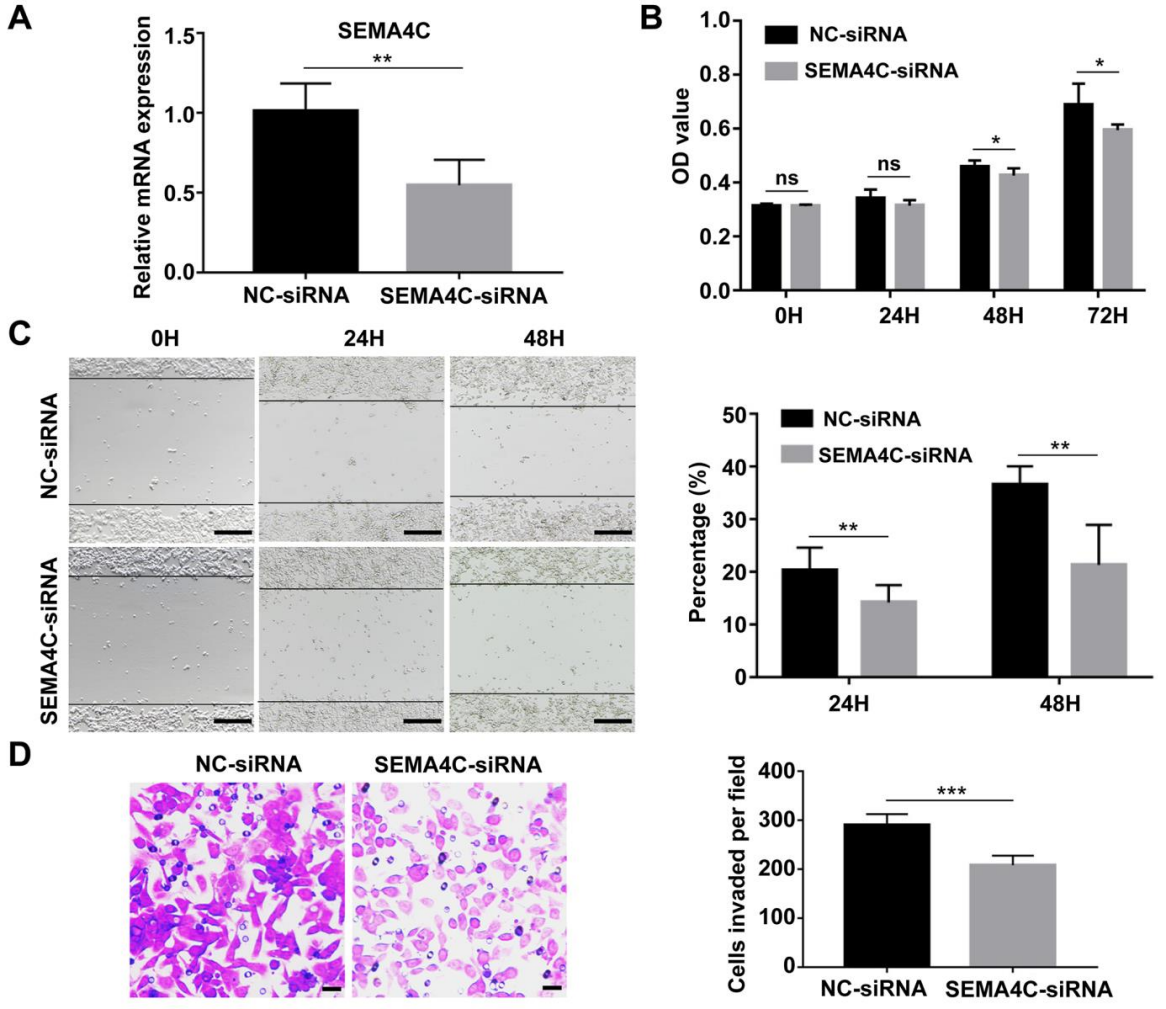
**SEMA4C silencing decreases proliferation, migration, and invasion of the LoVo cells.** (**A**) QRT-PCR analysis shows the SEMA4C mRNA levels in the NC-siRNA- or SEMA4C-siRNA-transfected LoVo cells (human colon adenocarcinoma cell line). (**B**) CCK-8 assay results show the viability of control and SEMA4C-silenced LoVo cells. (**C**) Wound-healing assay results show the migration rates of control and SEMA4C-silenced LoVo cells. Note: Scale bars=100 μm. (**D**) Transwell invasion assay results show the invasiveness of control and SEMA4C-silenced LoVo cells. Note: Scale bars=10 μm. H indicates hour. ***, *P* < 0.001; **, *P* < 0.01; *, *P* < 0.05; ns, not significant.

Next, we investigated the effects of SEMA4C silencing in the LoVo cells (metastatic human colon adenocarcinoma cell line). SEMA4C silencing significantly reduced SEMA4C mRNA expression in the LoVo cells compared to the corresponding controls (Figure 9A). Furthermore, CCK-8 assay results showed that knockdown of SEMA4C significantly reduced the proliferation of LoVo cells (Figure 9B). Moreover, wound healing assay results revealed that SEMA4C silencing significantly reduced the migration of LoVo cells compared to the corresponding controls (Figure 9C). Transwell invasion assay results illustrated that SEMA4C silencing significantly reduced the invasiveness of the LoVo cells (Figure 9D). These data demonstrate that SEMA4C knockdown reduces the growth and progression of CRC cells.

### SEMA4C silencing inhibits EMT in the LoVo cells

**Figure 10:**
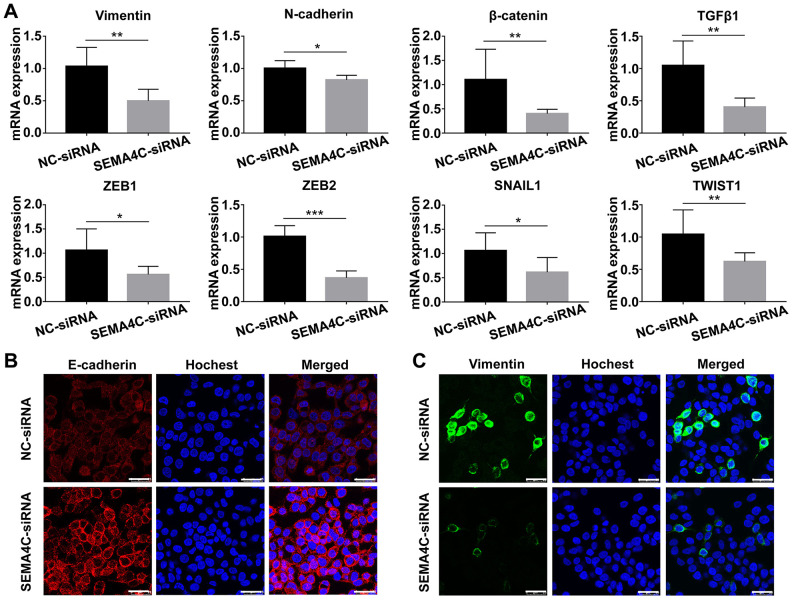
**SEMA4C silencing decreases the expression of EMT markers in the LoVo cells.** (**A**) QRT-PCR analysis shows the relative mRNA expression of EMT-related genes in the control and SEMA4C-silenced LoVo cells. ***, *P* < 0.001; **, *P* < 0.01; *, *P* < 0.05. (**B**, **C**) Representative immunofluorescence images (Scale bars=25 μm) show the levels of E-cadherin (red) (**B**) and Vimentin (green) (**C**) proteins in the control and SEMA4C-silenced LoVo cells. The nuclei are stained with Hoechst (blue).

We further analyzed the mRNA and protein expression of EMT-related markers in the control and SEMA4C-silenced LoVo cells. Quantitative real-time PCR (QRT-PCR) analysis showed that SEMA4C knockdown significantly reduced the mRNA levels of EMT markers (Vimentin, N-cadherin, β-catenin), pro-fibrogenic factor (TGFβ1), and EMT-inducing transcription factors including ZEB1, ZEB2, SNAIL1, and Twist-related protein 1 (TWIST1) (Figure 10A). Immunofluorescence results demonstrated that SEMA4C knockdown increased the expression of E-cadherin (epithelial marker), and decreased the expression of the Vimentin (mesenchymal marker) compared to the corresponding controls (Figure 10B). These data suggest that SEMA4C promotes EMT in the CRC cells.

## DISCUSSION

SEMA4C promotes oncogenic signaling in breast cancer, hepatocellular cancer, and osteosarcoma, it also regulates proliferation, migration, lymphangiogenesis, and metastasis of cancer cells [14, 16–18, 20, 22]. However, the prognostic significance of SEMA4C in colorectal cancer is not known. In the present study, we investigated the clinical significance of SEMA4C by analyzing the RNA-seq data from multiple CRC datasets and assessed SEMA4C protein expression by analyzing the immunohistochemistry data from the CRC samples in a TMA. Our study showed that SEMA4C mRNA and protein expression was significantly upregulated in the CRC tissues compared to the adjacent noncancerous colorectal tissues. High expression of SEMA4C positively correlated with the advanced pathologic stage and metastasis. Kaplan-Meier survival curve analysis revealed that the overall survival of CRC patients with high SEMA4C expression was significantly shorter than those with low SEMA4C expression. Univariate and multivariate analyses demonstrated that SEMA4C expression was an independent prognostic factor for CRC patients. High expression of SEMA4C correlates with poor prognosis of cervical cancer [16], breast cancer [14, 23], lung cancer [15] and HCC [17] patients. Our results also suggest that high SEMA4C expression indicates poor prognosis of CRC patients.

In this study, we also investigated the SEMA4C-related pathways that promote CRC growth and progression. PPI network, GO, and KEGG pathway analyses exhibited that SEMA4C and SEMA4C-related proteins could modulate chemotaxis, cell morphogenesis and differentiation, focal adhesion, cancer-related proteoglycans, oncogenic signaling pathways, VEGF signaling pathway, adherens junctions, and the ErbB signaling pathway. Moreover, functional enrichment analysis revealed that SEMA4C promoted EMT in the CRC cells. Furthermore, high SEMA4C expression positively correlated with lymph node metastasis, distant metastasis, and poor overall survival of CRC patients. These data suggest that SEMA4C is a potential prognostic predictor in CRC patients. High SEMA4C levels in breast cancer correlate with tumor progression and poor prognosis [14]. Gurrapu et al. showed that SEMA4C participates in reverse signaling and promotes metastasis by inducing genome-wide gene expression reprogramming and phenotypic plasticity in the invasive cancer cells [23]. Our findings indicate that SEMA4C plays an important role in colorectal cancer invasion and metastasis.

To gain further insights into the biological pathways regulated by SEMA4C during CRC progression, we performed GSEA using the RNA-seq data from the COAD and READ cohorts from the TCGA database. The results displayed significant enrichment of gene signatures related to EMT in the CRC patients with high SEMA4C expression. Moreover, CRC patients with high SEMA4C expression showed upregulation of the Wnt/β-catenin signaling pathway, which is required for the induction and maintenance of EMT [25]. EMT downregulates cell-cell adhesion, inhibits cell polarity and increases migration ability of the tumor cells [26–29]. Our study suggests that SEMA4C induces EMT through the activation of the Wnt/β-catenin signaling pathways.

Recent studies have identified four consensus molecular subtypes (CMS) with unique tumor biology and gene expression patterns in the CRC patients [6, 7]. CMS4 tumors are characterized by high expression of genes related to EMT, TGF signaling, matrix remodeling, and high stromal cell content [6, 7, 30]. CRC patients in the CMS4 group are associated with worse disease-free and overall survival rates [31]. Although CMS is a highly sensitive method for stratifying CRC patients based on tumor progression, it is currently difficult to perform routine clinical testing to determine tumor classification based on genome-wide transcriptome data because it is associated with high costs and requires larger tumor samples for testing. Therefore, there is an urgent need to identify specific biomarkers that can help classify CRC patients into specific CMS groups. Our study demonstrated that high SEMA4C expression correlated with the CMS4 subtype. ROC curve analysis revealed that SEMA4C expression could accurately distinguish CMS4 patients from CMS1-3 patients. Overall, our results confirm that high SEMA4C expression strongly correlates with the EMT phenotype, and support a critical role of SEMA4C in the CMS4 evolution process.

Epigenetic changes such as DNA methylation play a key role in regulating gene transcription and CRC development [32, 33]. Aberrant DNA methylation contributes to CRC progression by inactivating tumor suppressor genes or activating oncogenes [34, 35]. DNA methylation analysis of the TCGA-CRC samples showed that SEMA4C expression correlated negatively with the methylation of the 5’-UTR region in the SEMA4C gene. Furthermore, hypermethylation of the SEMA4C gene correlated with favorable prognosis in the CRC patients. We also did not observe significant copy number alterations or mutations of the SEMA4C gene in the CRC samples. These results demonstrate that DNA hypomethylation promotes CRC progression by increasing SEMA4C mRNA expression.

To further investigate the biological role of SEMA4C in the CRC cells, we silenced SEMA4C expression in the LoVo cells using SEMA4C-specific siRNAs. In the present study, SEMA4C silencing inhibited *in vitro* proliferation, migration, and invasion of LoVo cells by suppressing EMT. He et al. reported that overexpression of SEMA4C promotes the invasion and migration of CRC cells [36]. Quan et al. reported that downregulation of SEMA4C reduces the invasion and metastasis of cervical cancer cells by inhibiting EMT [22]. Moreover, SEMA4C knockdown inhibits the growth and EMT of non-small-cell lung cancer (NSCLC) cells [15]. Overall, our findings suggest that SEMA4C is a potential prognostic indicator of CRC progression. However, further investigations are required to confirm our findings and determine the clinical significance of SEMA4C.

In conclusion, we demonstrate that high SEMA4C expression correlates with CRC progression and CMS4 subtype by enhancing EMT. Furthermore, DNA hypomethylation upregulates SEMA4C expression in the CRC tissues. Overall, our results indicate that SEMA4C is an EMT-associated gene that effectively predicts prognosis of CRC patients and is a potential therapeutic target for CRC patients. Moreover, *in vitro* experiments confirm that SEMA4C may promote proliferation and metastasis of CRC cells by inducing the EMT process. Our study, therefore, offers a new understanding about CMS-specific features and will benefit subtype-specific therapies in CRC. The identification of molecularly distinct subtypes can provide distinct subsets of patients on which to conduct new treatments as well as clues for specific biological pathways to target for the molecular classification of CRC. We have extended the traditional pathology and clinical stage-based stratification of tumors, the potential future would enable us to stratify the treatments based on CMS-related analyses, not exposing CRC patients to toxic or targeted drugs from which they will derive no benefit. Such a future would have a higher probability of developing more precise treatments.

## MATERIALS AND METHODS

### CRC patient data from public databases

We obtained SEMA4C mRNA expression data from various cancer types using the Oncomine (https://www.oncomine.org/resource/login.html) database [37]. The threshold parameters were: *P*-value < 0.01; fold change ≥ 1.5; gene rank: top 10%; and data type: mRNA. We also analyzed relative SEMA4C mRNA expression levels in the tumor and normal tissue samples from different cancer types using the RNA-sequencing data from the TCGA and the Genotype-Tissue Expression (GTEx) projects with the interactive GEPIA (http://gepia.cancer-pku.cn) web server [38]. The pan-cancer expression profiles of SEMA4C were constructed based on the TCGA datasets using the Tumor Immune Estimation Resource (TIMER; https://cistrome.shinyapps.io/timer/) database [39]. We also downloaded the RNA-seq data of the TCGA-COAD and TCGA-READ cohorts from the Genomic Data Commons (GDC) and Application Programming Interface (API; https://gdc.cancer.gov/developers/gdc-application-programming-interface-api). The clinicopathological parameters of the TCGA-COAD and TCGA-READ patients were downloaded from the Firebrowse (http://firebrowse.org/) database. Only patients with fully characterized tumors and complete follow-up data were included. We also obtained mRNA expression and clinical data for 597 CRC samples and 51 normal samples from the TCGA database and analyzed the correlation between SEMA4C mRNA expression and clinicopathological characteristics. We downloaded the CMS subtyping data for the TCGA-CRC samples from the Colorectal Cancer Subtyping Consortium Synapse [6].

### Immunohistochemistry

We obtained a TMA of 83 paired colon cancer and adjacent normal colon tissue samples from Superbiotek, Shanghai, China (#COC160). The patient samples were obtained during surgery between May 2000 and February 2012. The diagnosis was based on histology. The follow-up information was available between October 2011 and September 2018. The survival times of the patients ranged from 2 to 146 months, with a median survival time of 69 months. The follow-up records were available for all cases. We also obtained clinicopathological information, including age, gender, T stage, lymphatic metastasis, distant metastasis, pathologic stage, overall survival time, tumor-free survival time, pretreatment serum CEA levels and pretreatment serum CA199 levels for all 83 patients. We performed this retrospective study using a commercial TMA for scientific research purposes only. The patient-sensitive clinical information was kept anonymous for this study.

For immunohistochemistry analysis, the TMA specimens were deparaffinized, hydrated and incubated with 3% H_2_O_2_ for 10 min to quench endogenous peroxidase activity. We then boiled the samples with citrate buffer (pH 6.0) for 90 sec in a steamer for antigen retrieval. The specimens were then blocked in 10% rabbit serum for 30 min and incubated overnight with the sheep anti-human primary SEMA4C antibody (#AF6125, R & D Systems, MN, USA) at 4^o^C. Then, we incubated the specimens with rabbit anti-goat horseradish peroxidase (HRP)-conjugated secondary antibody (#305-035-003, Jackson ImmunoResearch, PA, USA) for 30 min at 37^o^C. Then, the specimens were incubated with 3, 3’-diaminobenzidine (DAB) and counter-stained with hematoxylin.

The slides were digitally analyzed using the APERIO ScanScope (Leica Biosystems, Germany) and evaluated in the APERIO ImageScope (Leica Biosystems, Germany) with the positive pixel counting algorithm, which scored the staining as negative, weak-positive, medium, or strong. The histological score (H-score) for each sample was calculated with the following formula: 1×(% weak staining) + 2×(% moderate staining) + 3×(% strong staining). The values for the H-score ranged from 0 to 300. The slides were independently reviewed by two experienced pathologists who were blinded to the clinical parameters.

### Protein-protein interaction (PPI) network

We identified 50 SEMA4C-related proteins using the Search Tool for the Retrieval of Interacting Genes/Proteins (STRING; http://string-db.org/) database [40]. Then, we constructed a PPI network between SEMA4C and the 50 SEMA4C-interacting genes using the open-source Cytoscape platform (http://www.cytoscape.org) with optimal degree and closeness centrality parameters.

### Gene Ontology (GO) and the Kyoto Encyclopedia of Genes and Genomes (KEGG) functional enrichment analysis

The enriched GO terms for biological processes and cellular components [41], and the KEGG pathways [42] were analyzed for SEMA4C and 50 SEMA4C-associated genes using the Metascape platform (https://metascape.org/), which integrates multiple annotation datasets [43].

### Gene set enrichment analysis (GSEA)

The GSEA (http://www.broadinstitute.org/gsea) tool [44] was used to determine the differential enrichment of gene sets in the CRC patient samples belonging to high and low SEMA4C expression groups. The data for GSEA was accessible from The Cancer Genome Atlas (TCGA, https://cancergenome.nih.gov/) database and analyzed using standard settings of 1000 gene permutations. A normalized enrichment score (NES) was calculated for each gene set.

### Spearman’s correlation analysis

We analyzed the RNA sequencing expression data of CRC from the TCGA using the online database GEPIA (http://gepia.cancer-pku.cn/index.html) [38] to establish the correlation between the EMT-related genes and SEMA4C expression in CRC samples. The Spearman’s correlation co-efficient was evaluated for different genes and gene sets relative to SEMA4C expression.

### DNA methylation status and genomic alterations of the SEMA4C gene in CRC samples

We used the MEXPRESS (http://mexpress.be) tool [45, 46] to analyze and visualize the methylation status of the SEMA4C gene. The DNA methylation level of the CpG islands in the SEMA4C gene was expressed as a mean beta value. The data regarding frequency of copy number alterations and mutations in the SEMA4C gene for the CRC patients was obtained from the cBioPortal (https://www.cbioportal.org) database [47].

### Cell culture and siRNA transfections

The LoVo cell line (human colon adenocarcinoma cell line) was purchased from the BNBIO Company (Beijing, China) and cultured in F-12K medium (Hyclone, Logan, UT, USA) supplemented with 10% fetal bovine serum (FBS; Corning, NY, USA) and 1% penicillin–streptomycin (Gibco, Logan, UT, USA) in a humidified chamber at 37°C and 5% CO2. For siRNA transfections, LoVo cells were seeded in six-well plates and transfected with 200 nM SEMA4C-siRNA or NC-siRNA (Ribobio, Guangzhou, China) using Lipofectamine 3000 (Invitrogen, Carlsbad, CA, USA) according to the manufacturer’s instructions for 48 h.

### Quantitative real time PCR

Total RNA was extracted from the LoVo cells using the RNeasy kit (Beyotime, Shanghai, China, R0027) according to the manufacturer´s instructions. Then, we reverse transcribed 1 μg of total RNA from all samples using the SuperScript II reverse transcriptase (TaKaRa, Japan, RR047). Quantitative PCR analysis was performed using the SYBR Green Mix (TaKaRa, Japan, RR820) with the ABI 7900 HT Real-Time PCR system. The primer sequences for QRT-PCR were as follows: SEMA4C, 5′-ACTTATTGTGTCCCCGCGTA-3′, 5′-GCCCCATCAGAGCAATCGTT-3′; GAPDH, 5′-CTGACTTCAACAGCGACACC-3′, 5′-TGAGCTTGACAAAGTGGTCGT-3′; Vimentin, 5′-GCAGTTTTTCAGGAGCGCAA-3′, 5′-TCTTGTAGGAGTGTCGGTTGT-3′; N-cadherin, 5′-TTTGTGGTGGGAGCAGTAAGT-3′, 5′-CATGGTCTCATCCCCCAAGA-3′; β-catenin, 5′-TTGGAACCTTGTTTTGGACAGT-3′, 5′-AAGCATCGTATCACAGCAGGT-3′; TGFβ1, 5′-TGCCCATCGTCTACTACGTG-3′, 5′-TTGCAGGAGCGCACAATCAT-3′; ZEB1, 5′-CCCACTAGGAACAGGAACCAC-3′, 5′-CCCAACTTATGCCAGGCACC-3′; ZEB2, 5′-GGGCCTCTGTTTCAGGGTTG-3′, 5′-CTCGGTGCCATTTCTCTGACT-3′; SNAIL1, 5′-TAGAGTCTGAGATGCCCCGA-3′, 5′-AAATTGCCCGGGAAACAGGT-3′; TWIST1, 5′-ATCAAACTGGCCTGCAAAACC-3′, 5′-TTGCATTTTACCATGGGTCCTC-3′. Samples were run in triplicate and mRNA levels were expressed as a ratio relative to GAPDH expression.

### CCK-8 cell viability assay

Cell viability was assessed using the Cell Counting Kit 8 kit (CCK-8; DOJINDO, Kumamoto, Japan). Briefly, 1 × 10^4^ LoVo cells were seeded in 96-well plates and grown for 24, 48, and 72 h, respectively. Then, 10 μl CCK-8 reagent was added to each well and incubated for 2 h. The absorbance was measured at 450 nm using the microplate reader (PerkinElmer EnVision, MA, USA). Each sample was analyzed in triplicate. The experiments were repeated thrice.

### Wound-healing assay

LoVo cells were cultured in 6-well plates until they grew to 80-90% confluence. Then, the confluent monolayers were wounded by scratching with a 200 μl pipette tip. The cell debris was removed by rinsing with PBS. The cells were cultured for 48 h. Photographic images were taken at 0, 24, and 48 h after wounding using an inverted microscope (Nikon Eclipse, Ts2R, Nikon, Tokyo, Japan). The cell migration rate was calculated as the change in the wound area divided by the initial area of the wound.

### Transwell invasion assay

We seeded 1 × 10^5^ LoVo cells per well in 500 μl serum-free medium in the upper Transwell chambers (24-well insert, 8 μm, Corning, NY, USA) that were coated with the Matrigel (BD Biosciences, San Jose, CA, USA). We added 700 μl culture medium supplemented with 10% FBS as a chemoattractant to the bottom chamber. The Transwell chambers were incubated for 48 h in a humidified incubator at 37 °C and 5% CO_2_. Then, after removing the cells on the upper side of the Transwell, the invading cells on the underside of the Transwell were fixed with 4% paraformaldehyde for 30 min, stained with 0.1% crystal violet for 10 min, and counted under a light microscope using six randomly chosen fields to calculate the average number of invading cells for each sample.

### Immunofluorescence staining

LoVo cells were washed thrice with PBS, fixed with 4% paraformaldehyde for 15 min, permeabilized with 0.3% Triton X-100 for 30 min, and blocked with 5% BSA for 60 min. Then, the samples were incubated with primary antibodies against E-cadherin (1:100; Santa Cruz Biotechnology, CA, USA) and Vimentin (1:100; Boster, Wuhan, China) in 0.5% BSA overnight at 4 °C. The cells were then incubated with secondary antibodies (diluted 1:200) in 5% BSA for 1 h at room temperature followed by the nuclei-staining dye, Hoechst, for 10 min. The cells were washed thrice with PBS, and photographed with a confocal microscope (Leica SP8X, Germany).

### Statistical analysis

The data are expressed as mean ± standard deviation (SD). For continuous variables, comparisons between two groups were performed with the two-tailed unpaired or paired Student’s *t* test. The relationship between clinicopathological parameters and SEMA4C expression was assessed by the two-tailed Student’s *t* test or one-way analysis of variance (ANOVA). Survival analysis was performed using the survminer R package (version 3.5.0). Overall survival, progression-free survival and tumor-free survival rates were estimated using the Kaplan–Meier survival curves and the log-rank test. Cox proportional hazards regression was used to calculate the hazard ratios (HR). Multivariate Cox regression model was used to determine independent prognostic prediction factors. The association between SEMA4C expression and DNA methylation was evaluated by Spearman’s correlation analysis. Statistical analysis was performed using the SPSS 22.0 statistical software (SPSS Inc., Chicago, IL, USA). ROC curve analysis analyzed efficacy of differentiating CMS subtypes using SEMA4C expression levels. *P*-value < 0.05 was considered statistically significant.

### Ethical statement

This study was approved by the Ethics Committee of Institute of Materia Medica, Chinese Academy of Medical Sciences and Peking Union Medical College, and performed according to the principles of the Declaration of Helsinki. The requirement for informed consent was waived because of the retrospective nature of the study and the use of publicly available data.

## Supplementary Material

Supplementary Figures
