# Supplementary material for: High SEMA4C expression promotes the epithelial-mesenchymal transition and predicts poor prognosis in colorectal carcinoma
Source: Aging (Albany NY). 2020 Nov 7;12(21):21992–2018. doi: 10.18632/aging.104038 (PMC7695389; doi:10.18632/aging.104038)
Supplement: Supplementary Figures [file aging-12-104038-s001..pdf]

## SUPPLEMENTARY FIGURES

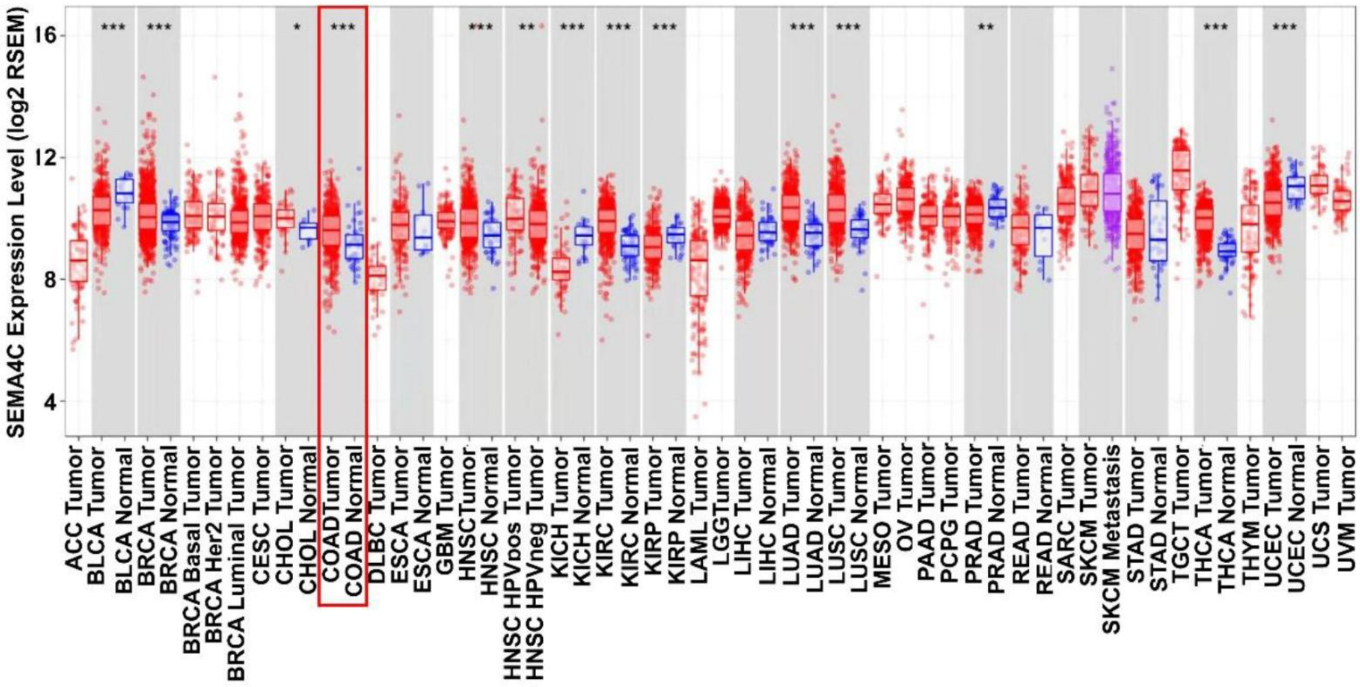


**Supplementary Figure 1. Pan-cancer expression profiling of SEMA4C mRNA levels in human cancers with the TIMER database.** The boxplots show the SEMA4C mRNA levels in the tumor (red) and normal (blue) tissue samples based on the TCGA data. SEMA4C was upregulated in 8 cancers (BRCA, CHOL, COAD, HNSC, KIRC, LUAD, LUSC, and THCA), and downregulated in 5 cancers (BLCA, KICH, KIRP, PRAD, and UCEC). Note: RSEM, RNA-Seq by expectation maximization; ***, $P < 0.001$; **, $P < 0.01$; *, $P < 0.05$; BRCA: Breast invasive carcinoma; CHOL: Cholangio carcinoma; COAD: Colon adenocarcinoma; HNSC: Head and Neck squamous cell carcinoma; KIRC: Kidney renal clear cell carcinoma; LUAD: Lung adenocarcinoma; LUSC: Lung squamous cell carcinoma; THCA: Thyroid carcinoma; BLCA: Bladder Urothelial Carcinoma; KICH: Kidney Chromophobe; KIRP: Kidney renal papillary cell carcinoma; PRAD: Prostate adenocarcinoma; UCEC: Uterine Corpus Endometrial Carcinoma.

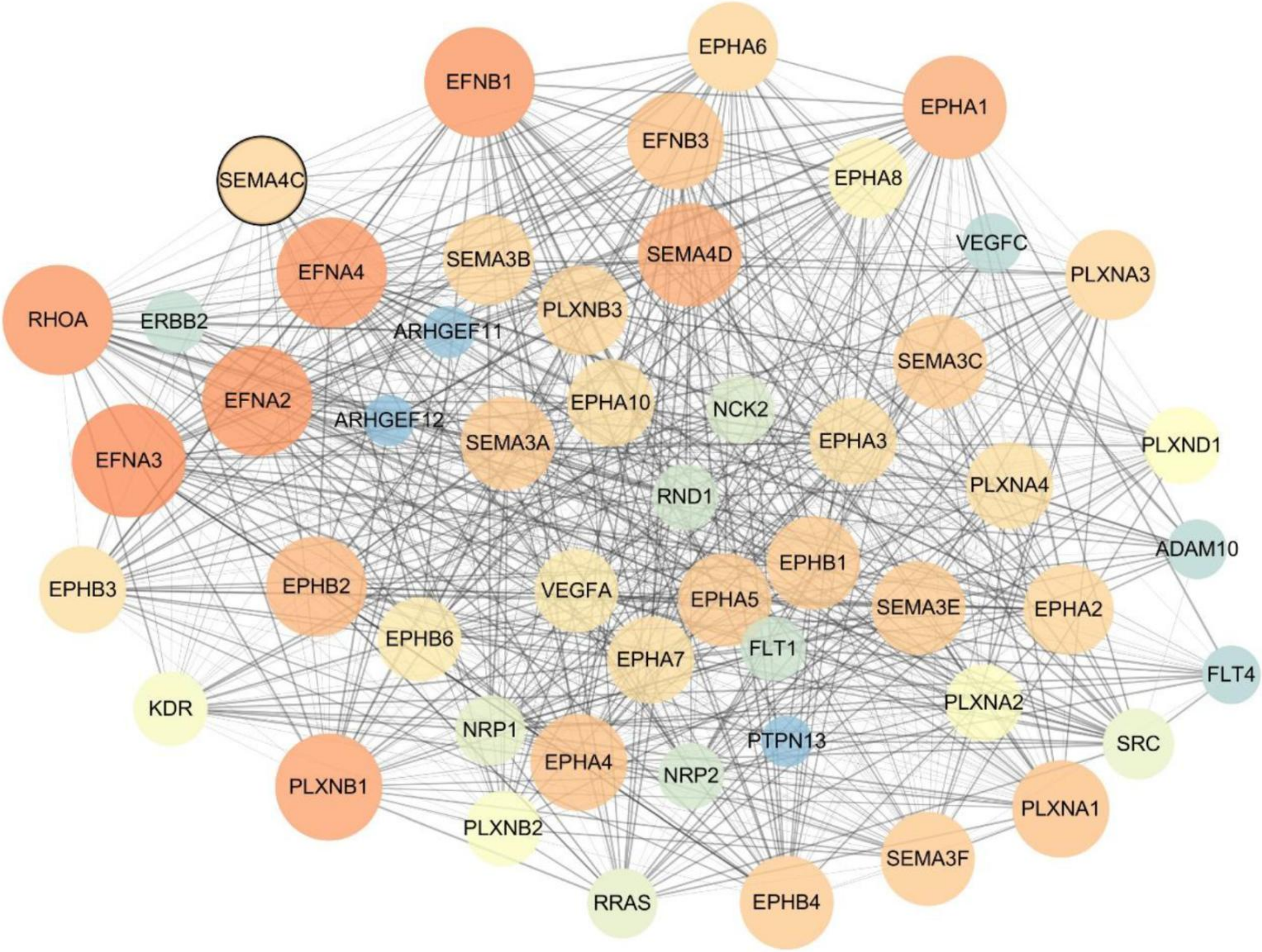


**Supplementary Figure 2. PPI network between SEMA4C and 50 SEMA4C-related genes.** The nodes denote proteins and the edges represent interactions between any two proteins in the PPI network.

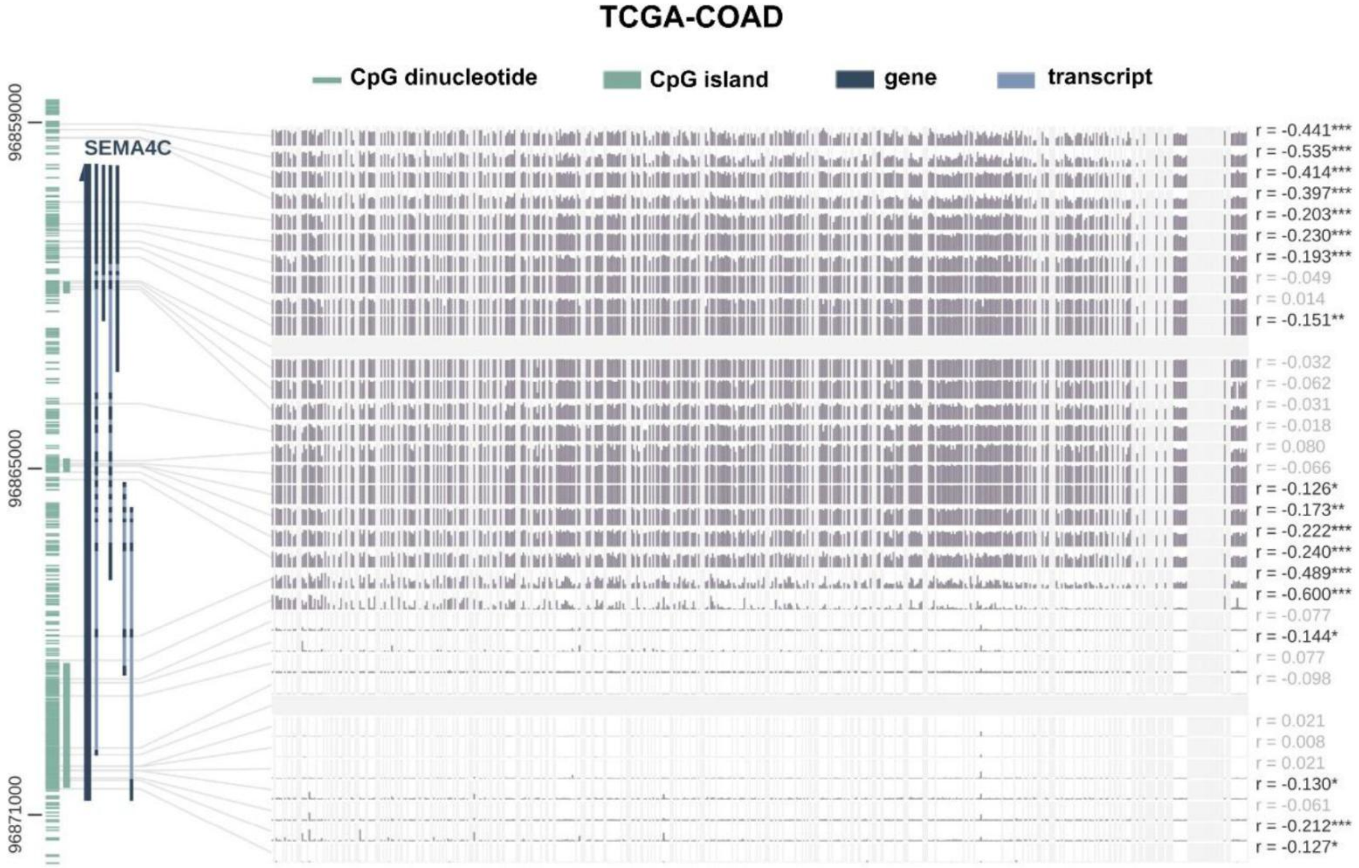


**Supplementary Figure 3. Association between SEMA4C expression and DNA methylation in the TCGA-COAD cohort using the MEXPRESS tool.** The correlation coefficients (r) are shown in the right panel; ***, $P < 0.001$; **, $P < 0.01$; *, $P < 0.05$.

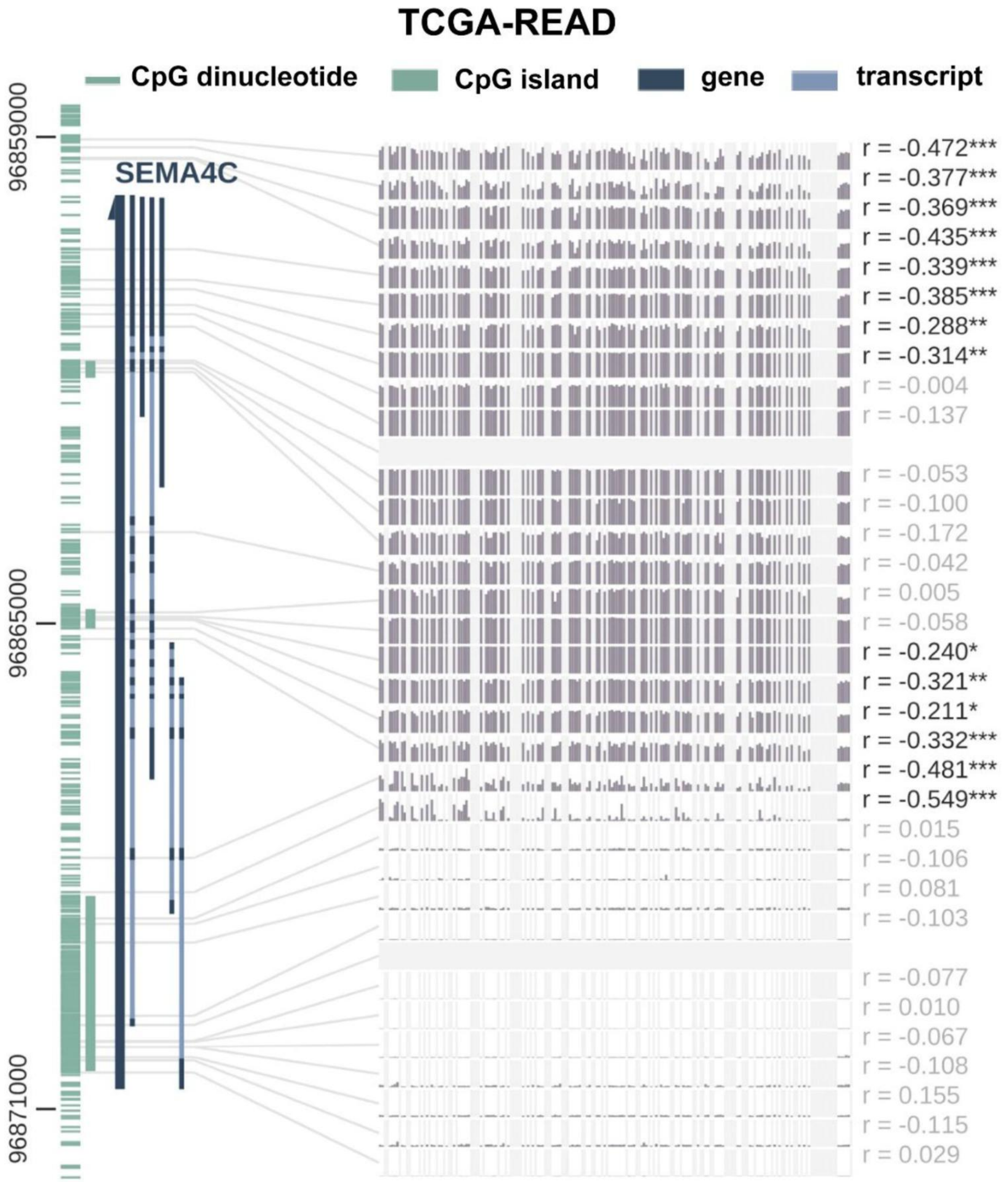


**Supplementary Figure 4. Association between SEMA4C expression and DNA methylation in the TCGA-READ cohort using the MEXPRESS tool.** The correlation coefficients (r) are indicated in the right panel; ***, $P < 0.001$; **, $P < 0.01$; *, $P < 0.05$.

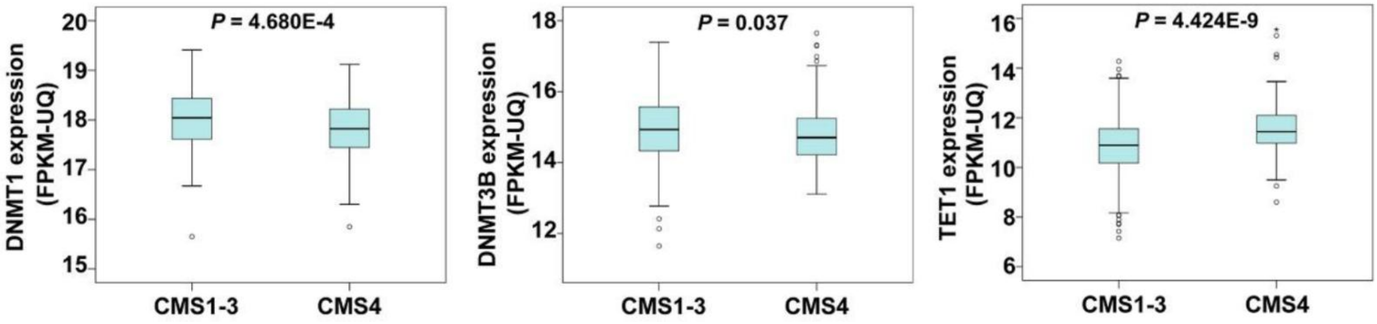


**Supplementary Figure 5. The mRNA levels of DNMT1, DNMT3B, or TET1 in the CMS1-3 and CMS4 molecular subtypes of the TCGA-CRC dataset.**

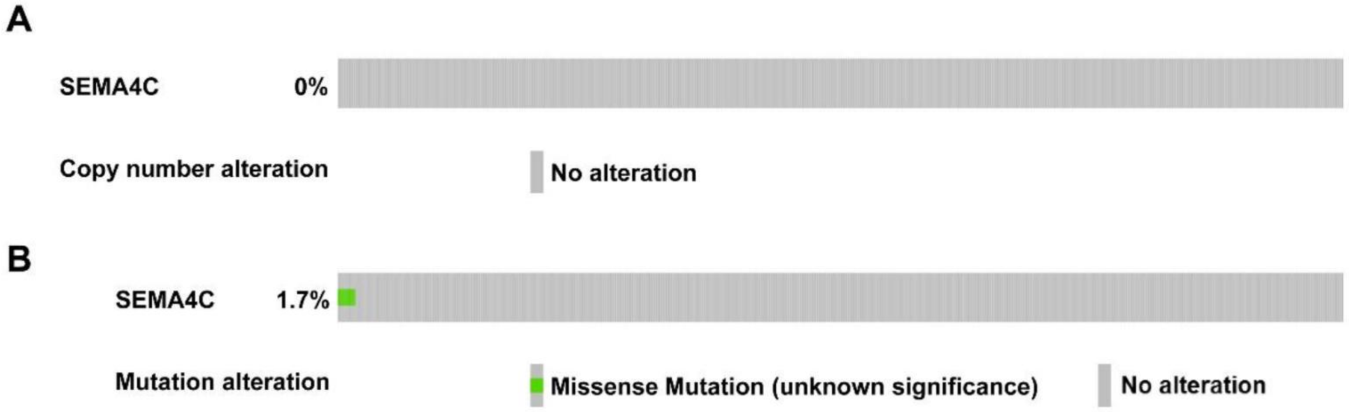


**Supplementary Figure 6. Analysis of SEMA4C gene alterations in the CRC samples using the cBioPortal.** The plots show the (**A**) copy number alterations and (**B**) mutations in the SEMA4C gene in the CRC samples (n=526) in the cBioPortal. Each gray box along the x-axis represents a single CRC patient.
